# Novel adamantane-nitrone hybrids: design, synthesis, and their multi-target neuroprotective effects against ischemic stroke

**DOI:** 10.3389/fphar.2026.1974944

**Published:** 2026-10-19

**Authors:** Hao Lu, Jiaxin Zheng, Ningning Luo, Chi Zhang, Xiao Liu, Xiaotong Wang, Yun Liu, Yong Ling, Xing Su

**Affiliations:** 1 Department of Neurosurgery, Affiliated Hospital of Nantong University, Nantong, China; 2 Nantong Key Laboratory of Small Molecular Drug Innovation, School of Medicine, Nantong University, Nantong, China; 3 Jiangsu Province Key Laboratory for Inflammation and Molecular Drug Target, School of Pharmacy, Nantong University, Nantong, China

**Keywords:** adamantane-nitrone hybrid, blood–brain barrier, ischemic stroke, multi-target neuroprotection, oxidative stress

## Abstract

**Introduction:**

Ischemic stroke involves interconnected pathological processes, including calcium overload, oxidative stress, apoptosis, and inflammation. This complexity challenges therapies directed at a single target, while limited blood–brain barrier (BBB) permeability can restrict drug delivery to the brain. This study aimed to develop hybrid compounds with favorable BBB permeability and multi-target neuroprotective activity.

**Methods:**

Four adamantane–nitrone hybrids (TA1–TA4) were synthesized and screened for cytotoxicity and neuroprotective activity, leading to the selection of TA1. TA1 was then evaluated for its effects on calcium overload, oxidative stress, mitochondrial function, and BBB permeability in vitro, and for therapeutic efficacy and related signaling pathways in MCAO mice. Single-dose safety was assessed separately.

**Results:**

TA1 showed the highest neuroprotective activity and lowest cytotoxicity among the four hybrids. It reduced calcium overload, scavenged ROS and ONOO^−^, and stabilized mitochondrial membrane potential. TA1 exhibited favorable BBB permeability (P_app_ = 6.73 × 10^−6^ cm/s) and ameliorated ischemia-induced barrier disruption. In MCAO mice, intravenous TA1 reduced cerebral infarct volume from 32.97% to 14.33%, outperforming the positive control TBN. It also alleviated brain edema and improved neurobehavioral outcomes. TA1 modulated the Bax/Bcl-2/caspase-3, Nrf2/HO-1, and NF-κB pathways. No overt abnormalities were observed in the measured hematological, biochemical, or histopathological indices 48 h after a single intravenous dose of 136 μmol/kg.

**Discussion:**

TA1 combines favorable BBB permeability with multi-target anti-apoptotic, antioxidant, and anti-inflammatory effects, supporting its further investigation as a candidate for ischemic stroke treatment.

## 1 Introduction

Ischemic stroke (cerebral infarction) is one of the leading causes of death and disability worldwide, imposing a heavy economic burden on society and healthcare systems (2021; Tsao et al., 2023; Xue et al., 2025). Its pathological mechanism involves a series of complex cascade reactions (Yang et al., 2025). In the ischemic core and penumbra, oxygen-glucose deprivation induces the pathological release of glutamate, which activates extrasynaptic NMDA receptors containing the NR2B subunit, leading to a massive influx of extracellular Ca^2+^ (Gardoni and Di Luca, 2006; Doyle et al., 2008; Liu et al., 2021; Yu et al., 2023; Xie et al., 2024; Fan et al., 2025). This excitotoxicity further activates neuronal nitric oxide synthase (nNOS) and induces mitochondrial dysfunction, generating abundant reactive oxygen species (ROS) and peroxynitrite (ONOO^−^) (Li et al., 2020; Anwar et al., 2025; Qin et al., 2025; Wang et al., 2025). Notably, these free radicals can damage glutamate transporter-1 (GLT-1) on astrocytes, further impeding glutamate clearance (Lu et al., 2020). Consequently, a vicious positive feedback loop forms between excitotoxicity and oxidative stress, ultimately exacerbating irreversible neuronal apoptosis. Therefore, developing neuroprotective strategies capable of simultaneously intervening at multiple key nodes within this cycle has become a crucial direction for novel drug discovery in stroke (Lo et al., 2005; Pacher et al., 2007; Manzanero et al., 2012; Lai et al., 2013; Liu et al., 2023; Liu et al., 2026).

In recent years, nitrone-based free radical scavengers have demonstrated highly promising potential as drug candidates in the field of stroke treatment (Floyd et al., 2013; Guo et al., 2013; Diez-Iriepa et al., 2022; Marco-Contelles, 2024). Among them, TBN (tetramethylpyrazine nitrone)—an innovative lead compound rationally designed by combining the nitrone moiety with tetramethylpyrazine (TMP), the active ingredient of the traditional Chinese medicine Ligusticum chuanxiong—has garnered significant attention due to its unique dual-functional mechanism. The nitrone functional group of TBN endows it with a robust capacity to trap superoxide anions (O_2_•^-^), hydroxyl radicals (•OH), and ONOO^−^, thereby effectively protecting mitochondria, alleviating oxidative damage, and providing substantial neuroprotective effects (Sun et al., 2012a; Sun et al., 2012b; Cao et al., 2021). Although TBN has exhibited a certain degree of free radical-scavenging ability and brain penetrability (Zhao et al., 2021), given the complex pathological network of stroke, there remains significant potential for improvement through structural modifications to further enhance the overall membrane permeability of the molecule while endowing it with multidimensional neuroprotective mechanisms (Zheng et al., 2024).

Concurrently, adamantane derivatives (such as memantine) have long established their unique clinical value in the treatment of central nervous system disorders (Czarnecka et al., 2021; Frost et al., 2024; Kim et al., 2024). Memantine can specifically block excessive extracellular Ca^2+^ influx and effectively inhibit neuronal excitotoxic injury by alleviating intracellular calcium overload (Lipton, 2006; Zhong et al., 2021; Phillips et al., 2026). In medicinal chemistry design, the adamantane scaffold, characterized by its highly lipophilic and sterically hindered three-dimensional symmetrical cage structure, is frequently employed as a functional carrier to significantly improve the blood–brain barrier (BBB) permeability of centrally acting drugs (Lipinski et al., 2001; Lipinski and Hopkins, 2004; Tetko et al., 2005; Wanka et al., 2013; Dane et al., 2025). Therefore, the adamantane skeleton serves not merely as a “functional carrier” to improve pharmacokinetic properties, but also as a pharmacophore with independent and verifiable neuroprotective activity.

Given the respective pharmacological advantages of TBN and adamantane compounds, alongside the limitations they face when applied individually, developing multi-target drugs that can simultaneously act on multiple pathological pathways has emerged as a highly rational drug discovery strategy to overcome the complex pathological networks of ischemic stroke (Cavalli et al., 2008; Ling et al., 2020; Zheng et al., 2026). To break the inextricably linked feedback loop between oxidative stress and excitotoxicity, structurally fusing the free radical-scavenging core of TBN (the nitrone-pyrazine pharmacophore) with the neuroprotective adamantane scaffold represents a highly promising and innovative hybridization approach (Viegas-Junior et al., 2007; Zhu et al., 2020). It is anticipated that this novel molecular hybridization design will not only enhance the therapeutic efficacy against ischemic stroke through the synergistic effects of the dual pharmacophores, but also ingeniously leverage the lipophilicity and cage structure of adamantane (Štimac et al., 2017) to further potentiate the BBB penetration efficiency of the molecules.

Based on the aforementioned tetramethylpyrazine/nitrone/adamantane fusion strategy, and by introducing various substituents onto the adamantane moiety, a series of novel tetramethylpyrazine/nitrone/adamantane hybrid molecules (**TA1**–**TA4**) were designed and synthesized in this study, followed by a systematic biological evaluation for ischemic stroke. Their cytocompatibility, protective effects against oxygen-glucose deprivation/reoxygenation (OGD/R)-induced neuronal injury, and anti-apoptotic activities were investigated. Concurrently, their antioxidant capacities—including the regulation of intracellular calcium homeostasis, scavenging of free radicals (ROS/ONOO^−^), and preservation of mitochondrial function—were analyzed, and their BBB penetrability as well as protective effects against hypoxia-induced loss of BBB integrity were preliminarily assessed. *In vivo*, using a mouse model of transient middle cerebral artery occlusion (MCAO), the efficacy of the active compounds in reducing infarct volume and ameliorating neurological deficits was evaluated. Furthermore, the potential molecular mechanisms underlying their regulation of apoptosis, oxidative stress, and neuroinflammation were preliminarily explored by detecting apoptosis-related proteins and changes in the Nrf2/HO-1 and NF-κB signaling pathways (Figure 1). Through comprehensive biological evaluations, we identified TA1 as a highly promising multi-target candidate. TA1 reduced ROS and ONOO^−^ accumulation, calcium overload, and mitochondrial depolarization *in vitro*, while showing favorable permeability. In MCAO mice, TA1 reduced infarct, exceeding the effect of TBN, and improved neurological outcomes. These effects were accompanied by increased Nrf2/HO-1 expression, restoration of the Bax/Bcl-2 balance, and reduced cleaved caspase-3 and NF-κB signaling, supporting TA1 as a multi-target candidate for ischemic stroke.

**FIGURE 1:**
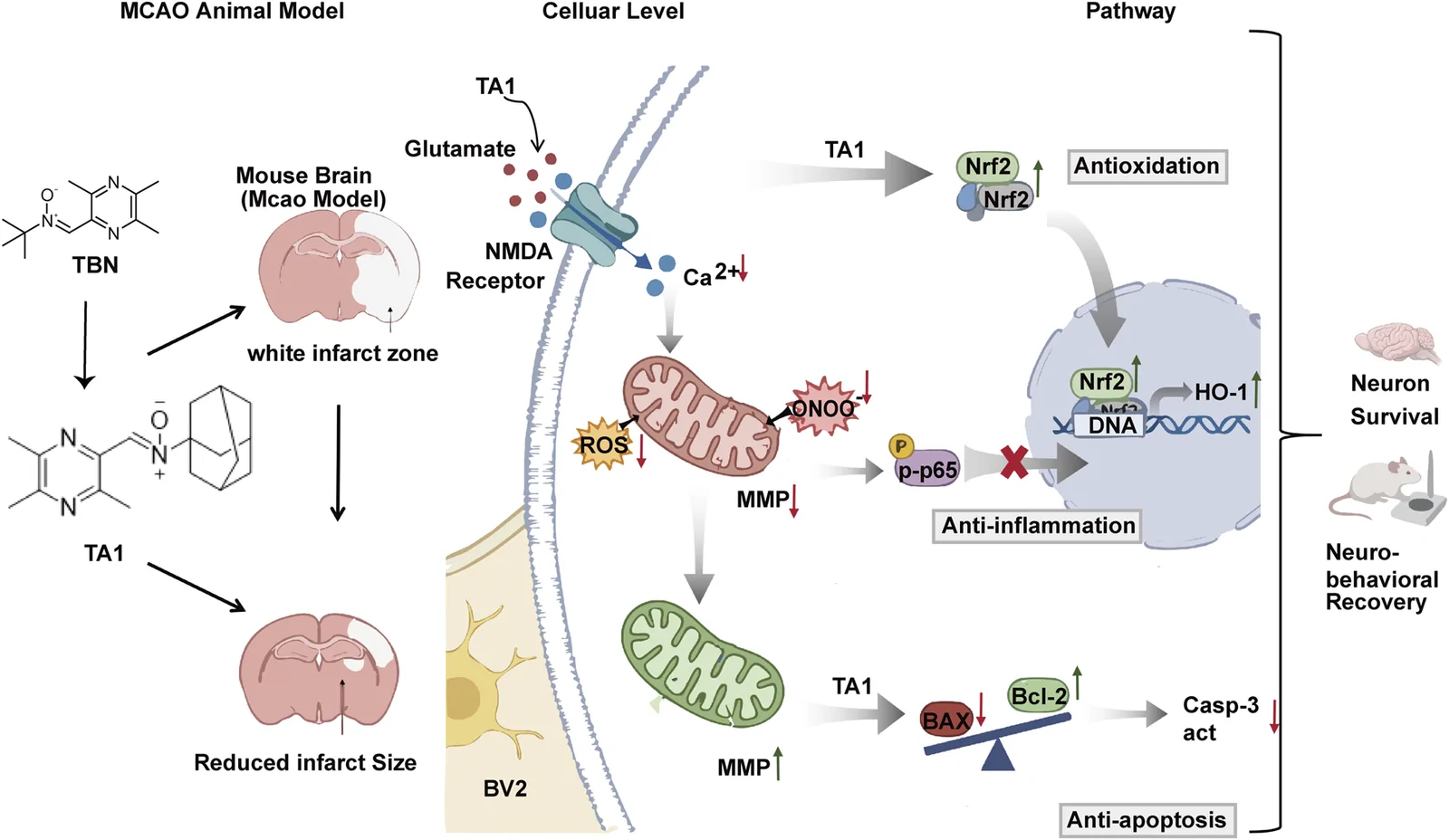
Schematic illustration of the multi-target neuroprotective mechanisms of **TA1** in ischemic stroke.

## 2 Materials and methods

### 2.1 Materials and general instrumentation

Unless otherwise specified, commercially available chemicals were obtained from Sigma-Aldrich (St. Louis, MO, USA) and Shanghai Titan Technology Co., Ltd. (Shanghai, China) and were used as received. Reaction progress was followed by thin-layer chromatography on silica gel plates (ca. 0.2 mm thickness; Shanghai J&K Scientific Ltd., Shanghai, China). Tetramethylpyrazine nitrone (TBN) was prepared in-house. tert-Butyl hydroperoxide (t-BHP) and sodium fluorescein were supplied by Aladdin (Shanghai, China), while 2,3,5-triphenyltetrazolium chloride (TTC) was obtained from Yeasen (Shanghai, China). The Cell Counting Kit-8 (CCK-8) and Annexin V-FITC/PI Apoptosis Detection Kit were obtained from KeyGen Biotech (Nanjing, China), and confocal culture dishes were supplied by NEST Biotechnology (Wuxi, China). JC-1, DCFH-DA, DHR123, and Fluo-3 AM were obtained from Beyotime Biotechnology (Shanghai, China), MedChemExpress (Monmouth Junction, NJ, USA), and Solarbio Science & Technology Co., Ltd. (Beijing, China), respectively, with DCFH-DA and DHR123 both purchased from MedChemExpress.

Structural characterization of the synthesized compounds was carried out using nuclear magnetic resonance (NMR) spectroscopy and high-resolution mass spectrometry (HRMS). ^1^H and ^13^C NMR spectra were recorded on a Bruker AV 400 MHz spectrometer (Bruker, Billerica, MA, USA), with tetramethylsilane (TMS) serving as the internal reference. Accurate mass measurements were obtained using an Agilent LC/MSD TOF mass spectrometer (Agilent Technologies, Santa Clara, CA, USA).

CCK-8 absorbance measurements and fluorescence measurements for the blood–brain barrier (BBB) permeability assay were performed using a Tecan Infinite 200 PRO multimode microplate reader (Tecan, Männedorf, Switzerland). Apoptotic cell populations were quantified by flow cytometry using a BD FACSAria III system (BD Biosciences, San Jose, CA, USA). Confocal fluorescence imaging of cells labeled with JC-1, DCFH-DA, or DHR123, as well as colocalization studies, was conducted using a Leica TCS SP8 confocal microscope (Leica Microsystems, Wetzlar, Germany).

### 2.2 Experiments

#### 2.2.1 Synthesis of TA1-TA4

(Z)-N-(adamantan-1-yl)-1-(3,5,6-trimethylpyrazin-2-yl)methanimine oxide (**TA1**).

The title compound **TA1** was prepared as follows. 3,5,6-Trimethylpyrazine-2-carbaldehyde (**1**, 150 mg, 1.0 mmol) and 1-adamantylamine (**2a**, 378 mg, 2.5 mmol) were placed in a Schlenk tube and dissolved in toluene (2 mL). The mixture was stirred under reflux overnight. After completion of the reaction, the insoluble solids were removed by filtration, and the filtrate was concentrated under reduced pressure to afford the colorless imine intermediate **3a**, which was then dissolved in dichloromethane (3 mL) in a reaction flask. A solution of m-CPBA (207 mg, 1.2 mmol) in dichloromethane was added dropwise to the flask at 0 °C. After the addition, the mixture was allowed to warm to room temperature and stirred vigorously for 5 h. Upon completion, the mixture was filtered with suction, and the filtrate was extracted with saturated aqueous sodium bicarbonate solution and dichloromethane. The organic layer was concentrated to give the imine N-oxide **5a**. The crude **5a** was further dissolved in acetonitrile (2 mL) and heated at reflux for 48 h. Finally, the resulting target compound **TA1** was purified by column chromatography (petroleum ether/ethyl acetate = 1:1, v/v), and the overall yield for the three-step sequence was 36%. ^1^H NMR (400 MHz, CDCI_3_) *δ* 7.67 (s, 1H, CH = N), 2.46–2.39 (m, 9H, 3 ✕ CH_3_), 2.20–2.16 (m, 9H, CH_2_, CH), 1.68 (s, 6H, 3 ✕ CH_2_). ^13^C NMR (101 MHz, DMSO-*d*_*6*_) *δ* 132.8, 131.0, 129.3, 128.3, 115.9, 84.9, 40.5, 35.3, 29.6. HRMS (ESI-MS) m/z: calcd for C_18_H_26_N_3_O [M + H]^+^ 300.2076, found 300.2081.

(Z)-N-(3,5-Dimethyladamantan-1-yl)-1-(3,5,6-trimethylpyrazin-2-yl)methanimine N-oxide (**TA2**).

3,5,6-Trimethylpyrazine-2-carbaldehyde (**1**, 150 mg, 1.0 mmol) and 3,5-dimethyladamantylamine (**2b**, 448 mg, 2.5 mmol) were dissolved in toluene (2 mL) and heated under reflux overnight. After completion of the reaction, the insoluble solids were removed by filtration, and the filtrate was concentrated under reduced pressure to afford the colorless imine intermediate **3b**, which was then dissolved in dichloromethane (3 mL) in a reaction flask. A solution of m-CPBA (207 mg, 1.2 mmol) in dichloromethane was added dropwise to the flask at 0 °C. After the addition, the mixture was allowed to warm to room temperature and stirred vigorously for 5 h. Upon completion, the mixture was filtered with suction, and the filtrate was extracted with saturated aqueous sodium bicarbonate solution and dichloromethane. The organic layer was concentrated to give the crude product **5b**, which was further dissolved in 3 mL of acetonitrile and heated at 80 °C under reflux for 48 h. After the reaction, the solution was concentrated under reduced pressure, and the residue was purified by column chromatography (petroleum ether/ethyl acetate = 1:1, v/v) to afford compound **TA2**. The overall yield for the three-step sequence was 35%. ^1^H NMR (400 MHz, CDCI_3_) *δ* 7.77 (s, 1H, CH = N), 2.54 (s, 3H, CH_3_), 2.51 (s, 3H, CH_3_), 2.47 (s, 3H, CH_3_), 2.37–2.34 (m, 1H, CH), 2.10 (d, *J* = 3.3 Hz, 2H, CH_2_), 1.93–1.85 (m, 4H, 2 ✕ CH_2_), 1.47–1.38 (m, 4H, 2 ✕ CH_2_), 1.26 (s, 2H, CH_2_), 0.96 (s, 6H, 2 ✕ CH_3_). ^13^C NMR (101 MHz, DMSO-*d*_*6*_) *δ* 152.0, 146.7, 142.0, 140.7, 73.1, 68.0, 67.7, 61.6, 54.5, 49.1, 46.5, 44.5, 37.7, 37.6, 30.5, 30.4, 30.3, 22.4, 13.6, 11.9. HRMS (ESI-MS) m/z: calcd for C_20_H_3_0N_3_O [M + H]^+^ 328.2389, found 328.2393.

(Z)-N-(3-Hydroxyadamantan-1-yl)-1-(3,5,6-trimethylpyrazin-2-yl)methanimine N-oxide (**TA3**).

3,5,6-Trimethylpyrazine-2-carbaldehyde (**1**, 150 mg, 1.0 mmol) and 3-hydroxyadamantylamine (**2c**, 418 mg, 2.5 mmol) were dissolved in toluene (2 mL) and heated under reflux overnight. After completion of the reaction, the insoluble solids were removed by filtration, and the filtrate was concentrated under reduced pressure to afford the colorless imine intermediate **3c**, which was then dissolved in dichloromethane (3 mL) in a reaction flask. A solution of m-CPBA (207 mg, 1.2 mmol) in dichloromethane was added dropwise to the flask at 0 °C. After the addition, the mixture was allowed to warm to room temperature and stirred vigorously for 5 h. Upon completion, the mixture was filtered with suction, and the filtrate was extracted with saturated aqueous sodium bicarbonate solution and dichloromethane. The organic layer was concentrated to give the crude product 5c, which was further dissolved in 3 mL of acetonitrile and heated at 80 °C under reflux for 48 h. After the reaction, the solution was concentrated under reduced pressure, and the residue was purified by column chromatography (petroleum ether/ethyl acetate = 1:1, v/v) to afford compound **TA3**. The overall yield for the three-step sequence was 32%. ^1^H NMR (400 MHz, DMSO-*d*_*6*_) *δ* 7.81 (s, 1H, CH = N), 4.73 (s, 1H, OH), 2.44 (s, 3H, CH_3_), 2.37 (s, 3H, CH_3_), 2.23 (s, 3H, CH_3_), 1.97–1.89 (m, 6H, 3 ✕ CH_2_), 1.53 (s, 4H, 2 ✕ CH_2_), 1.48–1.41 (m, 3H, CH_2_, CH), 1.16 (s, 1H, CH). ^13^C NMR (101 MHz, CDCl_3_) δ 151.7, 146.9, 86.1, 73.0, 69.6, 60.5, 48.0, 44.2, 43.5, 38.7, 36.4, 34.8, 31.5, 30.7, 29.7, 22.3, 13.6, 11.6. HRMS (ESI-MS) m/z: calcd for C_18_H_26_N_3_O_2_ [M + H]^+^ 316.2025, found 316.2028.

(Z)-N-(1-(Adamantan-1-yl)ethyl)-1-(3,5,6-trimethylpyrazin-2-yl)methanimine oxide (**TA4**).

3,5,6-Trimethylpyrazine-2-carbaldehyde (**1**, 150 mg, 1.0 mmol) and 1-(adamantan-1-yl)ethan-1-amine (**2d**, 448 mg, 2.5 mmol) were dissolved in toluene (2 mL) and heated under reflux overnight. After completion of the reaction, the insoluble solids were removed by filtration, and the filtrate was concentrated under reduced pressure to afford the colorless imine intermediate **3d**, which was then dissolved in dichloromethane (3 mL) in a reaction flask. A solution of m-CPBA (207 mg, 1.2 mmol) in dichloromethane was added dropwise to the flask at 0 °C. After the addition, the mixture was allowed to warm to room temperature and stirred vigorously for 5 h. Upon completion, the mixture was filtered with suction, and the filtrate was extracted with saturated aqueous sodium bicarbonate solution and dichloromethane. The organic layer was concentrated to give the crude product 5d, which was further dissolved in 3 mL of acetonitrile and heated at 80 °C under reflux for 48 h. After the reaction, the solution was concentrated under reduced pressure, and the residue was purified by column chromatography (petroleum ether/ethyl acetate = 1:1, v/v) to afford compound **TA4**. The overall yield for the three-step sequence was 30%. Starting material **2d** was used as a racemic mixture without enantiomeric separation, and **TA4** was obtained and evaluated as a racemic mixture. ^1^H NMR (400 MHz, CDCl_3_) *δ* 7.46 (s, 1H, CH = N), 2.36 (s, 3H, CH_3_), 2.21 (s, 3H, CH_3_), 2.17 (s, 3H, CH_3_), 2.15 (m, 1H, CH), 2.08 (d, *J* = 3.3 Hz, 2H, CH_2_), 1.84–1.75 (m, 4H, 2 ✕ CH_2_), 1.52–1.48 (m, 4H, 2 ✕ CH_2_), 1.26 (s, 2H, CH_2_), 0.86 (s, 6H, 2 ✕ CH_3_). ^13^C NMR (101 MHz, DMSO-*d*_*6*_) *δ* 152.0, 146.8, 142.0, 140.7, 72.9, 58.7, 51.6, 46.5, 38.6, 36.4, 36.3, 29.3, 29.3, 29.0, 22.3, 22.0, 21.8, 21.4, 13.6, 11.9. HRMS (ESI-MS) m/z: calcd for C_20_H_30_N_3_O [M + H]^+^ 328.2389, found 328.2391.

#### 2.2.2 Cell culture

PC12 rat pheochromocytoma cells, BV2 murine microglial cells, and SH-SY5Y human neuroblastoma cells were sourced from the Cell Bank of the Center for Excellence in Molecular Cell Science, Chinese Academy of Sciences (Shanghai, China). Routine cell maintenance was carried out in a humidified atmosphere containing 5% CO_2_ at 37 °C. High-glucose Dulbecco’s modified Eagle’s medium (DMEM; Gibco, Grand Island, NY, USA) containing 10% premium fetal bovine serum (NEWZERUM Ltd., Christchurch, New Zealand) and 1% penicillin–streptomycin (Solarbio, Beijing, China) was used as the complete culture medium.

#### 2.2.3 CCK-8 assay

The cytotoxicity of **TA1**, **TA2**, **TA3**, **TA4**, and TBN toward PC12 cells was evaluated using the CCK-8 assay. PC12 cells in the logarithmic growth phase were seeded in 96-well plates and cultured at 37 °C for 12 h. The cells were then treated with 100 µL of culture medium containing **TA1**, **TA2**, **TA3**, **TA4**, or TBN at various concentrations (0, 50, 100, and 200 µM). After 24 h, the CCK-8 reagent was added, and the cells were incubated for an additional 2 h. The absorbance at 450 nm was measured using a multimode microplate reader, and the cell viability was calculated from the absorbance values.

#### 2.2.4 Establishment of the OGD/R model

To establish the oxygen–glucose deprivation/reoxygenation (OGD/R) model, PC12, BV2, and SH-SY5Y cells were plated in 96-well plates (1 × 10^4^ cells/well) and allowed to grow for 24 h. The cells were subsequently subjected to OGD in a hypoxic incubator for 6 h. At the end of the deprivation period, OGD conditions were terminated by exchanging the medium for complete culture medium supplemented with the indicated concentrations of the test compounds. The cultures were then returned to normoxic conditions for an additional 24 h, after which the cell viability was quantified using the CCK-8 assay.

#### 2.2.5 JC-1 assay for mitochondrial membrane potential

BV2 cells were seeded in confocal dishes and cultured for 24 h to allow attachment. The culture medium was removed, and the cells were exposed to t-BHP at a final concentration of 150 μM for 1 h in a cell culture incubator. The medium was then removed, and the cells were gently washed twice with PBS. Subsequently, the cells were incubated for 2 h in serum-free medium containing **TA1** or TBN at various concentrations (0, 50, and 100 μM). After the medium was removed and the cells were washed with PBS, an appropriate volume of JC-1 staining solution was added. The cells were incubated in the dark for 20 min at 37 °C with 5% CO_2_. After incubation, the staining solution was removed, and the cells were washed with PBS and examined using a confocal microscope. The mitochondrial membrane potential was assessed based on the fluorescence intensity ratio of JC-1 aggregates (red fluorescence; λ_ex_/λ_em_ = 585/590 nm) to JC-1 monomers (green fluorescence; λ_ex_/λ_em_ = 514/529 nm).

#### 2.2.6 Measurement of intracellular reactive oxygen species (ROS)

BV2 cells were seeded in confocal dishes. After an initial incubation period, the medium was replaced with fresh complete culture medium, and t-BHP was added to a final concentration of 150 μM. The cells were incubated for an additional 1 h and then washed with PBS. Subsequently, the cells were treated with **TA1** or TBN at various concentrations (0, 50, and 100 µM) for 2 h. After the medium was removed and the cells were washed with PBS, they were incubated with 10 μM DCFH-DA for 30 min. The cells were then washed with PBS, counterstained with Hoechst 33342 to visualize the nuclei, and imaged using a confocal microscope.

#### 2.2.7 Measurement of intracellular peroxynitrite (ONOO^−^)

BV2 cells were seeded in confocal dishes. After an initial incubation period, the medium was replaced with fresh complete culture medium, and t-BHP was added to a final concentration of 150 μM. The cells were incubated for an additional 1 h and then washed with PBS. Subsequently, the cells were treated with **TA1** or TBN at various concentrations (0, 50, and 100 µM) for 2 h. After the medium was removed and the cells were washed with PBS, they were incubated with 10 μM DHR123 for 30 min. The cells were then washed with PBS, counterstained with Hoechst 33342 to visualize the nuclei, and imaged using a confocal microscope.

#### 2.2.8 Measurement of intracellular calcium (Ca^2+^)

BV2 cells were seeded in confocal dishes. After an initial incubation period, the medium was replaced with fresh complete culture medium, and t-BHP was added to a final concentration of 150 μM. The cells were incubated for an additional 1 h and then washed with PBS. Subsequently, the cells were treated with **TA1** or TBN at various concentrations (0, 50, and 100 µM) for 2 h. After the medium was removed and the cells were washed with PBS, they were incubated with 1 μM Fluo-3 AM for 60 min. The cells were then washed with PBS, counterstained with Hoechst 33342 to visualize the nuclei, and imaged using a confocal microscope.

#### 2.2.9 Establishment of the *in vitro* blood–brain barrier model

Mouse brain microvascular endothelial bEnd.3 cells were maintained in high-glucose DMEM containing 10% fetal bovine serum and 1% penicillin–streptomycin under standard culture conditions (37 °C, 5% CO_2_). A Transwell-based endothelial barrier was constructed using 12-well inserts (0.4 μm pore size; TCS016012, Guangzhou Jet Bio-Filtration Co., Ltd., Guangzhou, China). For each insert, 5.0 × 10^4^ bEnd.3 cells were plated on the apical side of the membrane, with 0.5 and 1.5 mL of complete medium added to the apical and basolateral compartments, respectively. The medium was refreshed every other day, and the cultures were maintained for 7 days to allow the formation of a confluent endothelial monolayer.

Barrier formation was monitored by measuring the transendothelial electrical resistance (TEER) using an epithelial voltohmmeter. The TEER values were corrected by subtracting the resistance of a cell-free insert and multiplying the resulting resistance by the effective membrane area. Monolayers were used for subsequent experiments when the TEER had reached a stable plateau and was ≥40 Ω cm^2^. Before permeability measurements, the culture medium was removed, and both compartments were gently washed twice with prewarmed Hanks’ balanced salt solution (HBSS). Unless otherwise specified, all permeability experiments were performed at 37 °C.

#### 2.2.10 Evaluation of the apparent BBB permeability of TA1 and TBN

The bEnd.3 monolayer model described in Section 2.2.9 was used to compare the apical-to-basolateral permeability of **TA1** and TBN. After equilibration with prewarmed HBSS for 30 min, **TA1** or TBN was added to the apical compartment at a final concentration of 300 µM in 0.5 mL of HBSS, while the basolateral compartment contained 1.5 mL of fresh HBSS. At 0.5, 1, and 2 h, 200 µL samples were collected from the basolateral compartment and immediately replaced with an equal volume of prewarmed HBSS to maintain a constant receiver volume. Samples were analyzed using a validated high-performance liquid chromatography (HPLC) method, and compound concentrations were calculated from the corresponding calibration curves.

The cumulative amount of compound transported into the basolateral compartment was corrected for the amount removed during previous sampling. The apparent permeability coefficient (P_app_) was calculated using the following equation:

$$P_{app}=\frac{dQ/dt}{A\times C_{0}}$$

where dQ/dt is the steady-state transport rate of the compound across the monolayer, A is the effective surface area of the Transwell membrane, and C_0_ is the initial compound concentration in the apical compartment. P_app_ is expressed in cm/s. Each condition was evaluated using three independent monolayers.

#### 2.2.11 OGD/R-induced BBB injury and sodium fluorescein permeability assay

The bEnd.3 monolayers established as described in Section 2.2.9 were used to evaluate the protective effects of **TA1** and TBN against oxygen–glucose deprivation/reoxygenation (OGD/R)-induced barrier disruption. For OGD, the culture medium in both compartments was replaced with glucose-free DMEM that had been pre-equilibrated under hypoxic conditions. The plates were placed in a hypoxia chamber containing 1% O_2_, 5% CO_2_, and 94% N_2_ at 37 °C for 6 h. Control monolayers were maintained for the same period in glucose-containing medium under normoxic conditions. At the end of OGD, the glucose-free medium was replaced with complete glucose-containing DMEM, and the cells were returned to normoxic culture conditions. **TA1** or TBN was added at the beginning of reoxygenation, and the monolayers were incubated for an additional 24 h.

After treatment, the medium was removed, and the apical and basolateral compartments were washed twice with prewarmed HBSS. Sodium fluorescein was added to the apical compartment at a final concentration of 10 μg/mL in 0.5 mL of HBSS, and 1.5 mL of HBSS was added to the basolateral compartment. Following incubation at 37 °C for 45 min, samples were collected from the basolateral compartment. The fluorescence intensity was measured using a fluorescence microplate reader at excitation and emission wavelengths of 485 and 535 nm, respectively. An increase in basolateral fluorescence indicated increased paracellular permeability and disruption of monolayer integrity.

The relative permeability was normalized to the normoxic control group and calculated using the following equation:

$$Relative\;permeability\left(\%\right)=\left(\frac{Mean\;fluorescence\;intensity\;of\;each\;group}{Mean\;fluorescence\;intensity\;of\;the\;control\;group}\right)\times 100$$

#### 2.2.12 Animals

ICR mice were supplied by the Experimental Animal Research Center of Nantong University (Nantong, China), and C57BL/6J mice were purchased from Sibeifu (Beijing) Biotechnology Co., Ltd. (Beijing, China). All mice were maintained in a specific pathogen-free (SPF) facility with regulated ambient temperature and a controlled light–dark cycle, with food and water available *ad libitum*. All experimental procedures involving animals were performed in compliance with institutional guidelines for laboratory animal care and use. The experimental protocol was reviewed and approved by the Animal Ethics Committee of Nantong University (Approval No. S20210925-003).

To achieve the predefined target sample sizes for all experimental assessments, a total of 163 mice were ultimately utilized in this study. The total number of mice subjected to the experiments in each group was as follows: Sham (n = 30), MCAO (n = 45), MCAO + TBN 136 μmol/kg (n = 29), MCAO + TA1 67 μmol/kg (n = 28), and MCAO + TA1 136 μmol/kg (n = 31). To prevent potential interference among different experimental procedures, separate cohorts of mice were independently used for the various outcome assessments. Specifically, individual cohorts were designated for TTC staining (n = 6 per group), brain edema measurement (n = 6 per group), long-term neurological and behavioral evaluations (n = 6 per group), Western blot analysis (n = 3 per group), and histopathological examinations (n = 6 per group). Additionally, two separate groups of mice (n = 3 per group) were utilized for systemic safety evaluations.

Throughout the experimental period, additional mice were continuously supplemented to replace those that died or failed to meet the inclusion criteria. Specifically, 7 mice from the initial MCAO modeling cohort were excluded prior to subsequent drug allocation due to surgical failures, such as accidental subarachnoid hemorrhage or the absence of typical neurobehavioral deficits. Furthermore, 15 mice (Sham: 0, MCAO: 11, MCAO + TBN: 2, MCAO + TA1 67 μmol/kg: 1, MCAO + TA1 136 μmol/kg: 1) died during the ischemia/reperfusion period due to severe stroke complications. Only the surviving animals that successfully met the inclusion criteria were included in the final data analyses.Therapeutic effects of TA1 against ischemic stroke *in vivo*.

Mice were randomly assigned to five experimental groups. In the Sham group, the relevant vessels were exposed and isolated without filament insertion, followed by the intravenous administration of saline. Animals in the MCAO group underwent MCAO surgery and received saline intravenously at the onset of reperfusion. The remaining MCAO-operated mice received TBN (136 μmol/kg), **TA1** (67 μmol/kg), or **TA1** (136 μmol/kg) intravenously immediately upon reperfusion and were designated as the MCAO + TBN, MCAO + **TA1** (67 μmol/kg), and MCAO + **TA1** (136 μmol/kg) groups, respectively. The experimental endpoint was set at 24 h after reperfusion.

Cerebral infarction was evaluated by 2,3,5-triphenyltetrazolium chloride (TTC) staining. At the designated endpoint, the mice were anesthetized, and the brains were carefully harvested and positioned in a brain matrix. After cooling at −20 °C for 30 min, each brain was cut into consecutive 2-mm-thick coronal slices. The sections were immersed in 2% TTC and incubated at 37 °C for 30 min in the dark. Following staining, the slices were fixed in 4% paraformaldehyde for 24 h. Digital images of the stained sections were analyzed using ImageJ software (National Institutes of Health, Bethesda, MD, USA) to quantify the infarcted regions.

For histopathological examination, brain tissues from a separate cohort were harvested 24 h after reperfusion and immersed in 4% paraformaldehyde for 24 h. The fixed tissues were dehydrated, paraffin-embedded, sectioned, and subjected to hematoxylin and eosin (H&E) staining. Histological evaluation was performed using coded sections by investigators unaware of the treatment allocation. The investigators responsible for assessment were not involved in the experimental design or tissue preparation, thereby maintaining blinding throughout the analysis.

Before behavioral assessment, the mice were allowed to acclimatize to the testing environment for 30 min. Each behavioral session was video-recorded for subsequent offline analysis. Between individual animals, the testing apparatus was cleaned with 70% ethanol and left to dry for at least 5 min. Behavioral outcomes were independently evaluated by two investigators who remained blinded to the group allocation.

Neurological function was evaluated 48 h after reperfusion using the modified Zea Longa scale by two blinded investigators. Deficits were graded from 0 to 4 as follows: 0, normal neurological function; 1, incomplete extension of the contralateral forelimb; 2, circling toward the contralateral side during locomotion; 3, a tendency to fall toward the contralateral side; and 4, absence of spontaneous locomotor activity accompanied by a reduced level of consciousness.

Forelimb-use asymmetry was examined using the cylinder test. Each mouse was allowed to move freely within a transparent glass cylinder for 5 min while its spontaneous forelimb activity was recorded. Video recordings were reviewed at 0.25× playback speed by two investigators blinded to the experimental groups. Wall contacts involving the ipsilateral forelimb, contralateral forelimb, or both forelimbs simultaneously were recorded separately. Impaired forelimb use was calculated as follows:

$$Impaired\;forelimb\;use\left(\%\right)=\left[\frac{N_{contralateral}}{N_{ipsilateral}+N_{contralateral}}\right]\times 100$$

For the corner turn test, mice were introduced into an apparatus composed of two walls forming a 30° corner with a 5-mm opening at the vertex. Each animal completed 10–15 trials, with an intertrial interval of 30 s. The direction of turning after entry into the corner was recorded, whereas turns not associated with a rearing movement were excluded from the analysis. The corner turn (CT) score was calculated using the following equation:

$$CT\;score=\left[\frac{R}{R+L}\right]\times 100$$

where R and L denote the numbers of rightward and leftward turns, respectively.

#### 2.2.13 Western blot assay

Proteins were extracted from ischemic penumbra tissues using ice-cold lysis buffer supplemented with protease and phosphatase inhibitors. Following lysis on ice, the samples were centrifuged at 13,000 rpm for 30 min at 4 °C to remove insoluble material, and the resulting supernatants were retained for analysis. Protein concentrations were determined using a BCA assay kit (Servicebio, Wuhan, China). Equivalent amounts of protein were resolved by sodium dodecyl sulfate–polyacrylamide gel electrophoresis (SDS-PAGE) and electrotransferred onto polyvinylidene difluoride (PVDF) membranes (Servicebio, Wuhan, China). After blocking, the membranes were probed overnight at 4 °C with primary antibodies against Bax, Bcl-2, cleaved caspase-3, Nrf2, HO-1, p-IκBα, p-p65, histone H3, and β-actin (all from Servicebio, Wuhan, China), with β-actin serving as the loading control. The membranes were then incubated with the appropriate horseradish peroxidase (HRP)-conjugated secondary antibodies (Servicebio, Wuhan, China) for 2 h at room temperature. Immunoreactive bands were developed using an enhanced chemiluminescence (ECL) detection system (Servicebio, Wuhan, China), and band intensities were quantified by densitometry using ImageJ software (National Institutes of Health, Bethesda, MD, USA).

#### 2.2.14 Safety assessment

The systemic safety profile of **TA1** was evaluated following a single intravenous administration *via* the tail vein at the onset of reperfusion. At 48 h after dosing, blood was collected from the orbital sinus for hematological and serum biochemical analyses. Hematological parameters included red blood cell count (RBC), hemoglobin (HGB), and platelet count (PLT), whereas serum biochemical indices included alanine aminotransferase (ALT) and aspartate aminotransferase (AST) for hepatic function, and urea (UREA) and creatinine (CREA) for renal function. After blood collection, the heart, liver, spleen, lungs, and kidneys were harvested and fixed for histopathological evaluation by hematoxylin and eosin (H&E) staining.

#### 2.2.15 Statistical analysis

Statistical analyses were conducted using GraphPad Prism 9.5 (GraphPad Software). Quantitative data are presented as mean ± standard deviation (SD), with the sample size (n) specified in the corresponding figure legends or Methods. Groupwise comparisons were performed using one-way analysis of variance (ANOVA). A P value <0.05 was considered statistically significant. Statistical significance in the figures is denoted as follows: *P < 0.05, **P < 0.01, ***P < 0.001, and ****P < 0.0001; ns indicates no statistically significant difference.

## 3 Results and discussion

### 3.1 Synthesis of TA1-TA4

The target compounds **TA1**–**TA4** were synthesized *via* a sequential three-step procedure (Scheme 1). Initially, 3,5,6-trimethylpyrazine-2-carbaldehyde (1) was condensed with various substituted adamantylamine 2a-d in refluxing toluene to afford the corresponding imine intermediates 3a-d, which were subsequently oxidized with m-chloroperoxybenzoic acid (m-CPBA) in dichloromethane at room temperature to get imine N-oxides 5a-d. After rapid extraction and concentration, the crude oxides were further heated in refluxing acetonitrile to induce configurational isomerization for 48 h. The resulting target compounds **TA1**–**TA4** were finally purified by column chromatography to >95% purity (Supplementary Figures S13–S16), and their structures were fully characterized by ^1^H NMR, ^13^C NMR, and HRMS (Supplementary Figures S1–S12).

**SCHEME 1:**
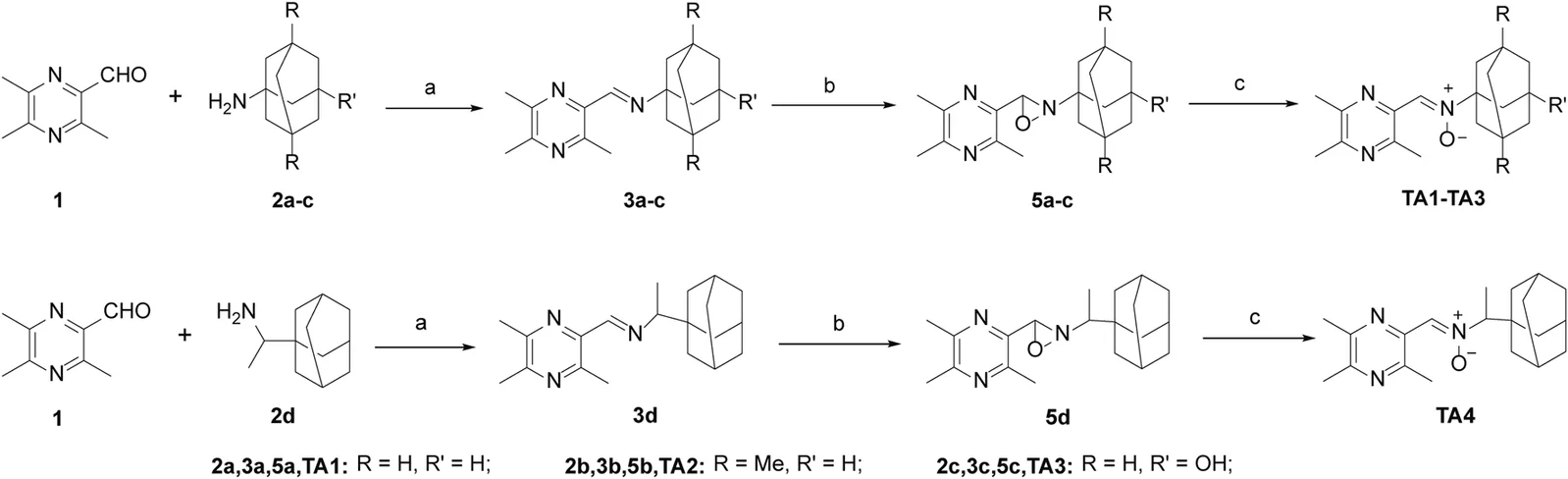
Synthetic route to **TA1**–**TA4**. **(a)** Toluene, reflux, 12 h; **(b)** m-CPBA, CH_2_Cl_2_, 0 °C to rt, 5 h; **(c)** Acetonitrile, reflux, 48 h, 30%–36% for three-step procedure.

### 3.2 *In Vitro* biocompatibility evaluation of TA1

The cytotoxicity of compounds **TA1**–**TA4** and the positive control TBN against PC12 and SH-SY5Y cells was assessed using the CCK-8 assay (Figure 2A). Quantitative cell viability results indicated that **TA1** exhibited minimal cytotoxicity at concentrations up to 100 μM. At a treatment concentration of 100 μM, the survival rates of both neural cell lines remained above 90%. Conversely, equivalent concentrations of **TA2**, **TA3**, **TA4**, and the positive control TBN induced varying degrees of reduction in cell viability (Figures 2B,C).

**FIGURE 2:**
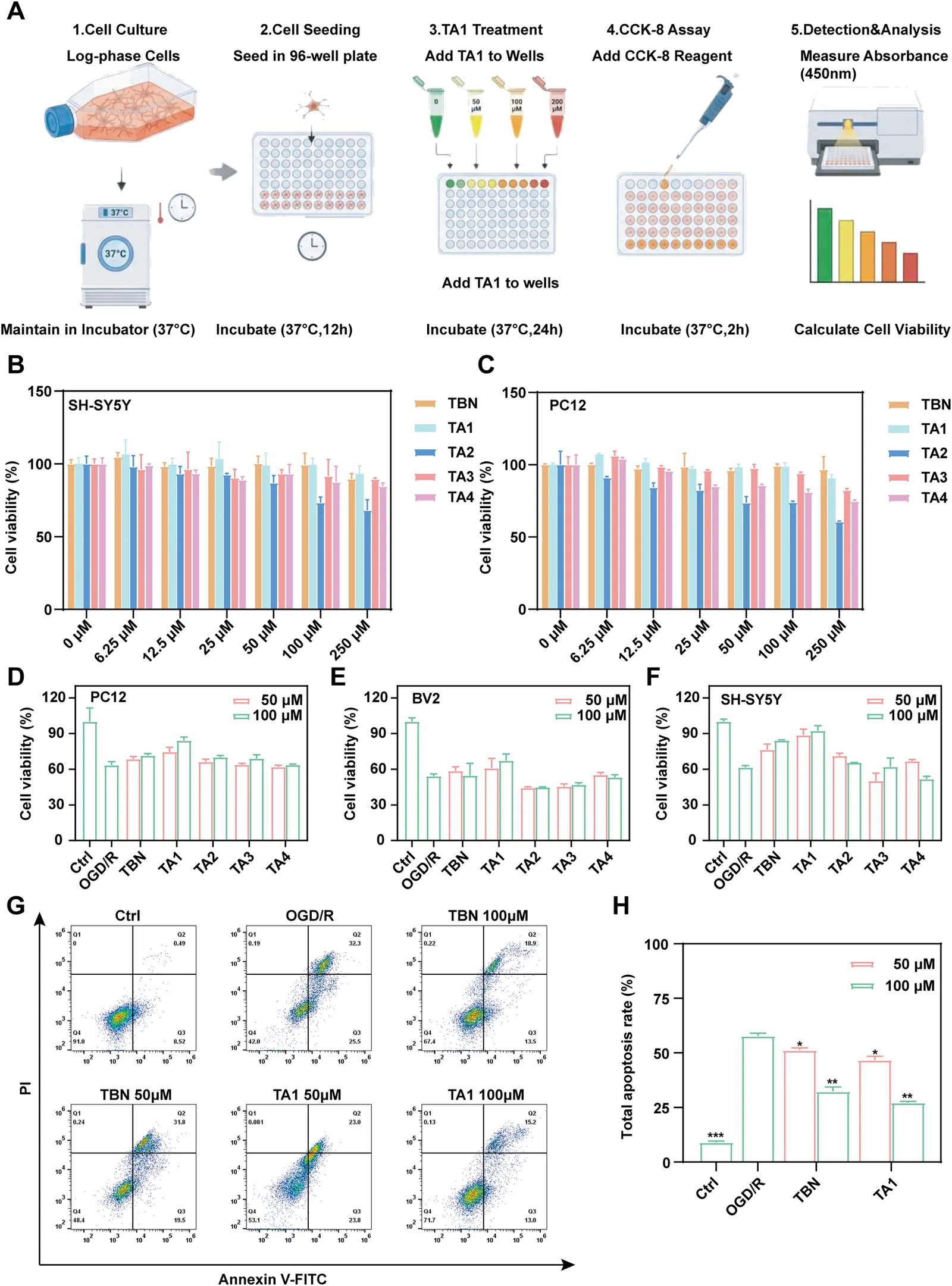
*In vitro* biocompatibility of **TA1** and its neuroprotective effects against OGD/R-induced injury in neural cells. **(A)** Schematic illustration of the experimental design for the CCK-8 cell viability assay. **(B,C)** Cell viability of SH-SY5Y **(B)** and PC12 **(C)** cells treated with varied concentrations (6.25–250 μM) of TBN, **TA1**, **TA2**, **TA3**, and **TA4** for 24 h under normoxic conditions to assess inherent cytotoxicity. **(D–F)** Cell viability of PC12 **(D)**, BV2 **(E)**, and SH-SY5Y **(F)** cells subjected to oxygen-glucose deprivation/reoxygenation (OGD/R) challenge, followed by treatment with 50 and 100 μM of the specified compounds for 24 h. **(G)** Representative flow cytometry plots utilizing Annexin V-FITC/PI dual staining to assess the anti-apoptotic effects of TBN and **TA1** (50 and 100 μM) in BV2 microglia following OGD/R exposure. **(H)** Quantitative analysis of the total apoptosis rate (combining early and late apoptosis quadrants) in BV2 cells derived from the flow cytometry data. Data are expressed as mean ± SD (n = 3). *p < 0.05, **p < 0.01, and ***p < 0.001 versus the OGD/R model group.

### 3.3 TA1 inhibits programmed cell death and alleviates OGD/R-induced neuronal injury

To examine the cytoprotective activity of **TA1** under ischemia/reperfusion-like conditions, BV2, PC12, and SH-SY5Y cells were subjected to oxygen-glucose deprivation/reoxygenation (OGD/R). OGD/R reduced the viability of all 3 cell types to approximately 50% of the corresponding control values. Treatment with **TA1** at 50 or 100 μM improved cell survival in a concentration-dependent manner, with 100 μM elevating the survival rates to over 75% (Figures 2D–F). At equivalent concentrations, the restorative effect of 100 μM **TA1** on ischemia-injured cells was higher than that of **TA2**, **TA3**, **TA4**, and TBN.

Furthermore, Annexin V-FITC/PI double-staining flow cytometry was employed to quantitatively detect the apoptosis rate in BV2 cells (Figure 2G). The elevated apoptosis level induced by OGD/R was decreased following **TA1** treatment in a concentration-dependent manner (50–100 μM). Comparative experiments confirmed that the efficacy of 100 μM **TA1** in reducing cellular apoptosis was greater than that of an equivalent dose of TBN (Figure 2H).

### 3.4 TA1 antagonizes t-BHP-induced oxidative stress injury and calcium overload

The cytoprotective activity of **TA1** against oxidative damage was investigated using BV2, PC12, and SH-SY5Y cells exposed to tert-butyl hydroperoxide (t-BHP). **TA1** (50 and 100 μM) restored the viability of oxidatively damaged cells, with 100 μM **TA1** exhibiting the most prominent restorative effect, which was superior to that of the equivalent dose of TBN (Figures 3A–C). In the t-BHP-induced oxidative damage model in BV2 cells, four fluorescent probes (Fluo-3AM, JC-1, DCFH-DA, and DHR123) were utilized in parallel to detect intracellular calcium homeostasis, mitochondrial function, and oxidative/nitrosative stress. Confocal fluorescence imaging revealed that t-BHP stimulation induced massive accumulation of ROS and ONOO^−^ (increased DCFH-DA and DHR123 fluorescence intensities) (Figures 3F,G,J,K), marked intracellular calcium overload (enhanced Fluo-3AM green fluorescence) (Figures 3D,H), and mitochondrial depolarization (increased JC-1 green/red fluorescence ratio) (Figures 3E,I). Concurrent treatment with **TA1** and TBN (50 and 100 μM) reversed these t-BHP-induced changes in a dose-dependent manner. Specifically, **TA1** demonstrated a greater capacity than TBN in reducing Fluo-3AM fluorescence, restoring the JC-1 ratio, and decreasing DCFH-DA and DHR123 fluorescence intensities.

**FIGURE 3:**
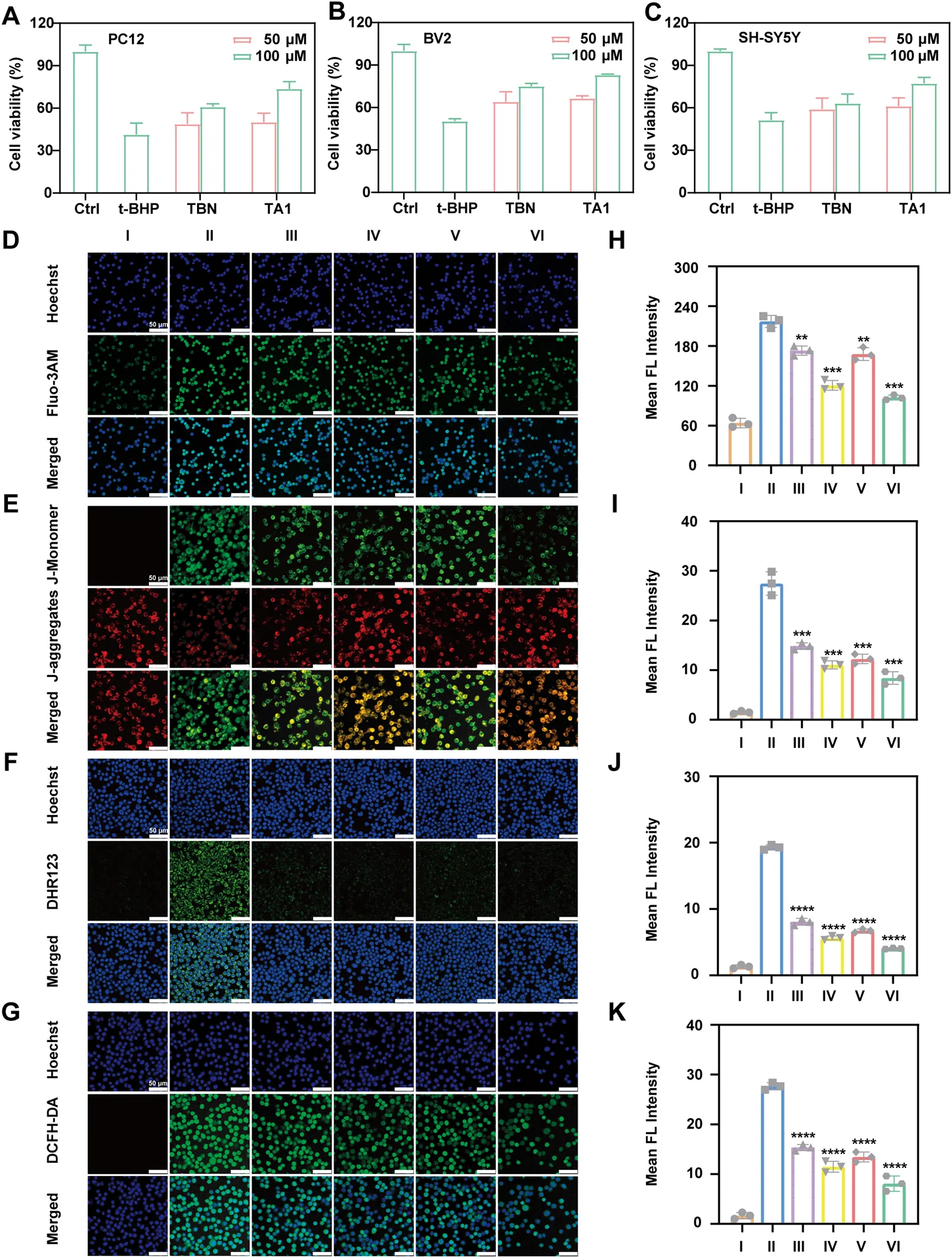
**TA1** protects neural cells against calcium overload and oxidative stress. **(A–C)** Cell viability of PC12 **(A)**, BV2 **(B)**, and SH-SY5Y **(C)** cells exposed to t-BHP followed by treatment with **TA1** or TBN (50 and 100 μM) (CCK-8 assay). The Roman numerals in panels **(D–K)** indicate the corresponding treatment groups: **(I)** Ctrl; II: t-BHP; III: TBN (50 μM); IV: TBN (100 μM); V: **TA1** (50 μM); VI: **TA1** (100 μM). **(D,H)** Representative images **(D)** and quantification **(H)** of intracellular Ca^2+^ levels detected by Fluo-3AM probe. **(E,I)** Representative images **(E)** and quantification **(I)** of JC-1 staining showing mitochondrial membrane potential (Δψm) in BV2 cells. **(F,J)** Representative images **(F)** and quantification **(J)** of intracellular ONOO^−^ levels detected by DHR123 probe. **(G,K)** Representative images **(G)** and quantification **(K)** of intracellular ROS levels detected by DCFH-DA probe. Nuclei were stained with Hoechst (blue). Data are shown as mean ± SD (n = 3). *p < 0.05, **p < 0.01, ***p < 0.001, and ****p < 0.0001 versus model group. Scale bar = 50 μm.

### 3.5 Evaluation of BBB permeability and protective efficacy against disruption

An *in vitro* blood–brain barrier (BBB) model using mouse brain microvascular endothelial cells (bEnd.3) was established to determine the apparent permeability coefficients (P_app_) of the compounds (Figure 4A). The P_app_ of the positive control TBN was 5.16 × 10^−6^ cm/s, while **TA1** exhibited a P_app_ of 6.73 × 10^−6^ cm/s (Figure 4B). In bEnd.3 cells subjected to OGD/R injury, cell viability declined to approximately 55%. Following **TA1** treatment, the cell survival rate was elevated, and this effect was superior to that of an equivalent dose of TBN (Figure 4C). Furthermore, in the *in vitro* Transwell BBB model, sodium fluorescein permeability in the OGD/R model group increased to approximately 219% of the normal control group. Treatment with TBN and **TA1** decreased the permeability to approximately 148% and 113%, respectively (Figure 4D).

**FIGURE 4:**
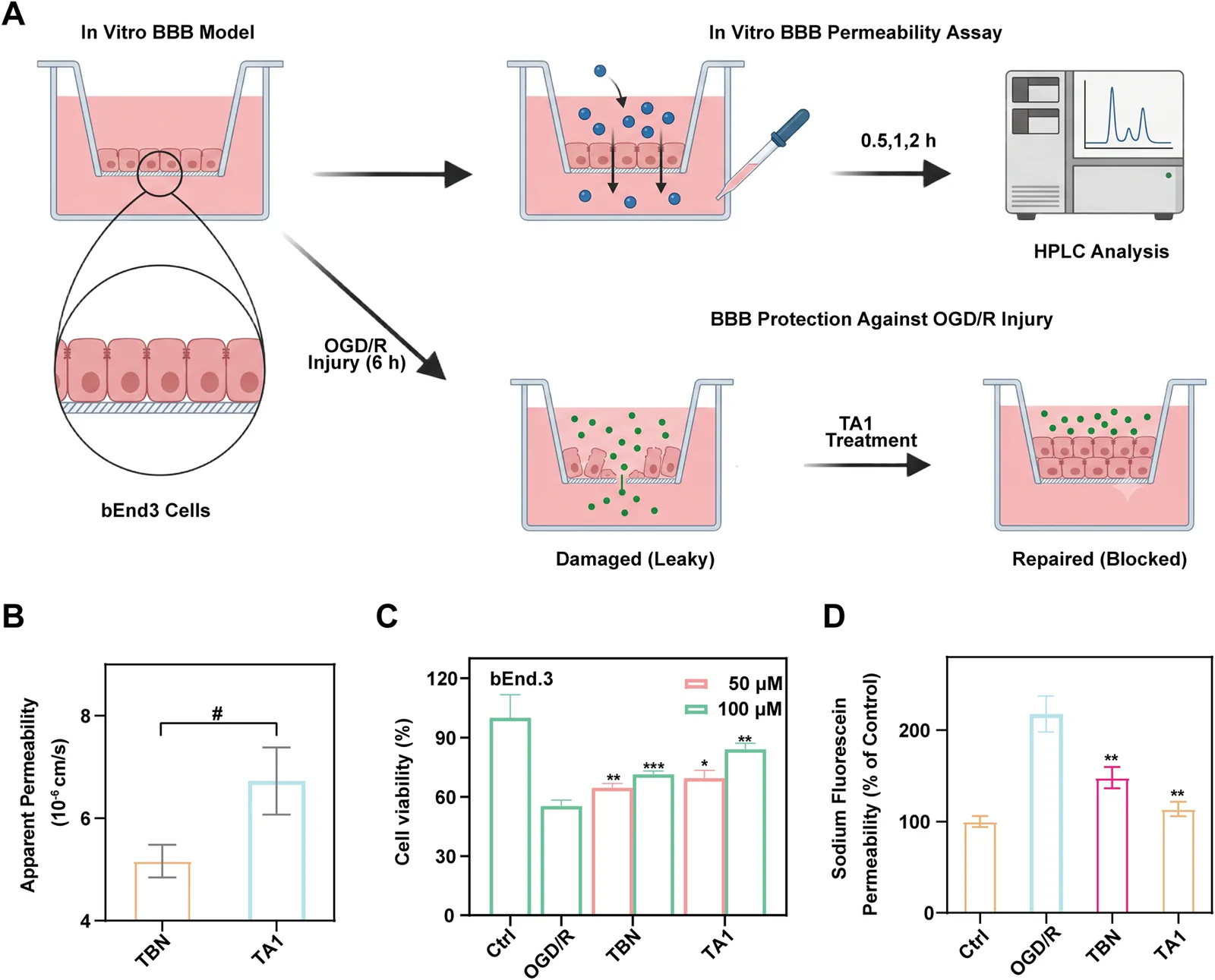
BBB permeability and protective effects of **TA1**. **(A)** Schematic illustration of the experiments in **(B,C)**, and **(D)**. **(B)** Apparent permeability coefficient (P_app_) of TBN and **TA1**. **(C)** Cell viability of bEnd.3 cells subjected to OGD/R challenge, followed by treatment with **TA1** or TBN (50, 100 μM) for 24 h (CCK-8 assay). **(D)** Sodium fluorescein permeability across the bEnd.3 monolayer after OGD/R. Data are expressed as mean ± SD (n = 3). *p < 0.05, **p < 0.01, ***p < 0.001, ****p < 0.0001 versus OGD/R model group; #p < 0.05 versus TBN-treated group.

### 3.6 Evaluation of the therapeutic efficacy of TA1 in ischemic stroke

The *in vivo* efficacy of TA1 was investigated in the mouse MCAO model (Figure 5A). TTC staining revealed that **TA1** administration decreased the cerebral infarct volume from approximately 32.97% in untreated MCAO mice to approximately 14.33%, a reduction greater than that achieved with TBN (Figures 5B,C). Cerebral edema was also lower in **TA1**-treated mice compared to untreated MCAO mice (Figure 5D). Histological examinations using H&E and Nissl staining showed prominent neuronal damage and loss of Nissl substance in the hippocampal (CA1, CA3, DG) and cortical regions of untreated MCAO mice. TBN partially improved these histological changes, whereas **TA1** produced a more pronounced preservation of neuronal architecture and Nissl substance in a dose-related manner (Figures 5E,F). Functional outcomes assessed 48 h after MCAO showed that **TA1** treatment improved performance across the Zea Longa score, cylinder test, and corner test compared to the MCAO group (Figures 5G–I).

**FIGURE 5:**
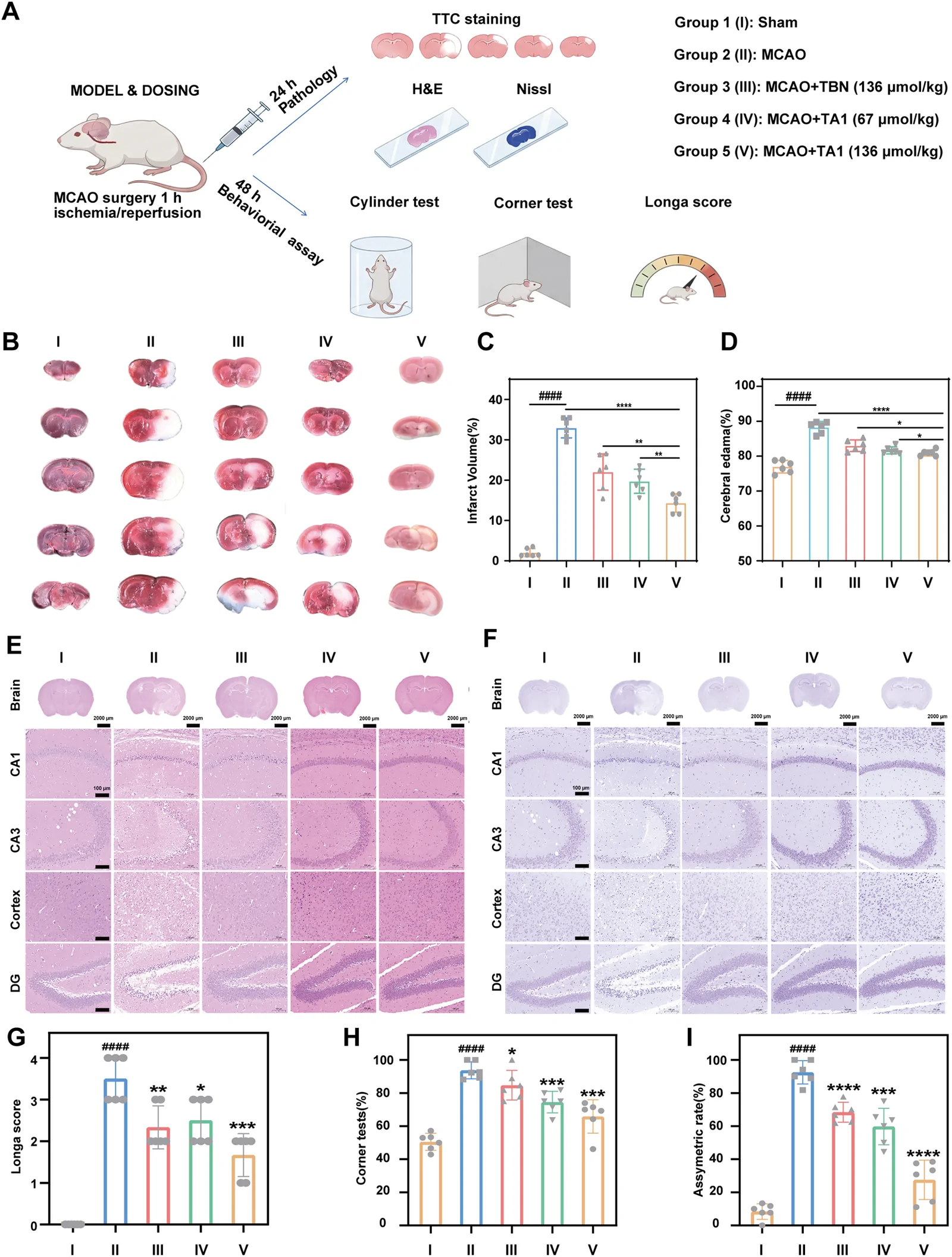
**TA1** reduces infarct volume, mitigates brain damage, and promotes neurological recovery in MCAO mice. **(A)** Schematic illustration of the *in vivo* experimental protocol for MCAO mice. **(B,C)** Representative images of TTC-stained brain sections **(B)** and quantitative analysis of the infarct volume **(C)** in mice from different groups. **(D)** Quantitative analysis of cerebral edema. **(E,F)** Representative H&E staining **(E)** and Nissl staining **(F)** images of the CA1, CA3, Cortex, and DG regions of the brain. Scale bars = 2000 μm (whole brain) and 100 μm (magnified regions). **(G–I)** Neurological and behavioral evaluations performed 48 h after reperfusion, including Zea Longa score **(G)**, Corner test **(H)**, and Asymmetric rate evaluated by the cylinder test **(I)**. Data are expressed as mean ± SD (n = 6). #p < 0.05, ##p < 0.01, ###p < 0.001, and ####p < 0.0001 versus the Sham group; *p < 0.05, **p < 0.01, ***p < 0.001, and ****p < 0.0001 versus the MCAO model group.

### 3.7 Effects of compound TA1 on Nrf2/HO-1, apoptosis, and NF-κB pathways in the ischemic penumbra of MCAO mice

Western blot analysis was performed on ischemic penumbra tissues. Compared with the Sham group, the relative expression levels of Nrf2 and HO-1 normalized to β-actin were decreased in the MCAO group (Figures 6A,E,F). **TA1** treatment restored these protein levels in a dose-dependent manner. High-dose **TA1** (136 μmol/kg) upregulated Nrf2 expression more significantly than the equimolar TBN control group. Regarding apoptosis-related proteins, the expression of Bax and cleaved caspase-3 was elevated, while Bcl-2 was reduced in the MCAO group compared to the Sham group (Figures 6B,G–J). **TA1** intervention reversed these alterations. High-dose **TA1** exhibited a stronger inhibitory efficacy on cleaved caspase-3 than the equimolar TBN group. For inflammatory markers, the expression levels of cytosolic p-IκBα and nuclear p-p65 were elevated in the MCAO group (Figures 6C,D,K). **TA1** treatment reversed these changes, with high-dose **TA1** demonstrating more robust ameliorative effects on p-IκBα and p-p65 than the equimolar TBN control group.

**FIGURE 6:**
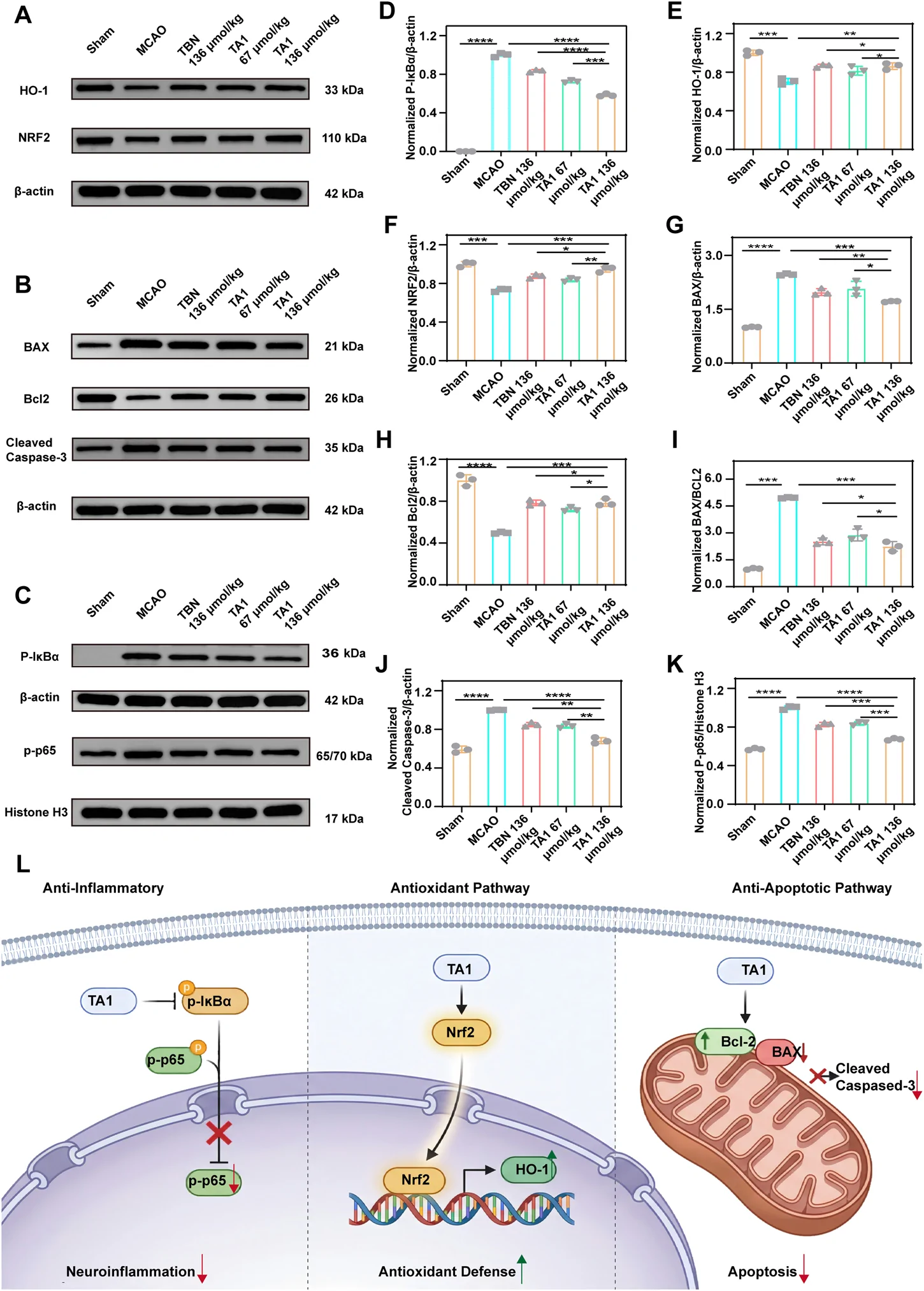
**TA1** suppresses apoptosis and neuroinflammation while activating the Nrf2/HO-1 antioxidant pathway in the ischemic penumbra of MCAO mice. **(A–C)** Representative Western blot images showing the expression levels of HO-1, Nrf2 **(A)**, apoptosis-related proteins Bax, Bcl-2, cleaved caspase-3 **(B)**, and p-IκBα, nuclear p-p65 **(C)** in the ischemic penumbra of mice from different groups. β-actin and Histone H3 were used as loading controls for cytosolic and nuclear proteins, respectively. **(D–F)** Quantitative analysis of the relative protein levels of p-IκBα **(D)**, HO-1 **(E)**, and Nrf2 **(F)** normalized to β-actin. **(G–J)** Quantitative analysis of the relative protein levels of Bax **(G)**, Bcl-2 **(H)**, Bax/Bcl-2 **(I)**, and cleaved caspase-3 **(J)** normalized to β-actin. **(K)** Quantitative analysis of the relative protein level of nuclear p-p65 normalized to Histone H3. **(L)** Schematic diagram illustrating the multi-target neuroprotective mechanisms of **TA1**. Data are expressed as mean ± SD (n = 3). *p < 0.05, **p < 0.01, ***p < 0.001, and ****p < 0.0001.

### 3.8 Safety evaluation of TA1

Blood, serum, and major organs were collected at 48 h post-administration (136 μmol/kg) for safety evaluation (Figure 7A). The levels of hepatic indicators (ALT, AST) and renal indicators (UREA, CREA) were within normal ranges (Figures 7B,C). Hematological parameters, including RBC, HGB, and PLT, remained stable (Figure 7D). Histopathological evaluation (H&E staining) of the heart, liver, spleen, lungs, and kidneys showed preserved structural integrity with no detected abnormalities (Figure 7E).

**FIGURE 7:**
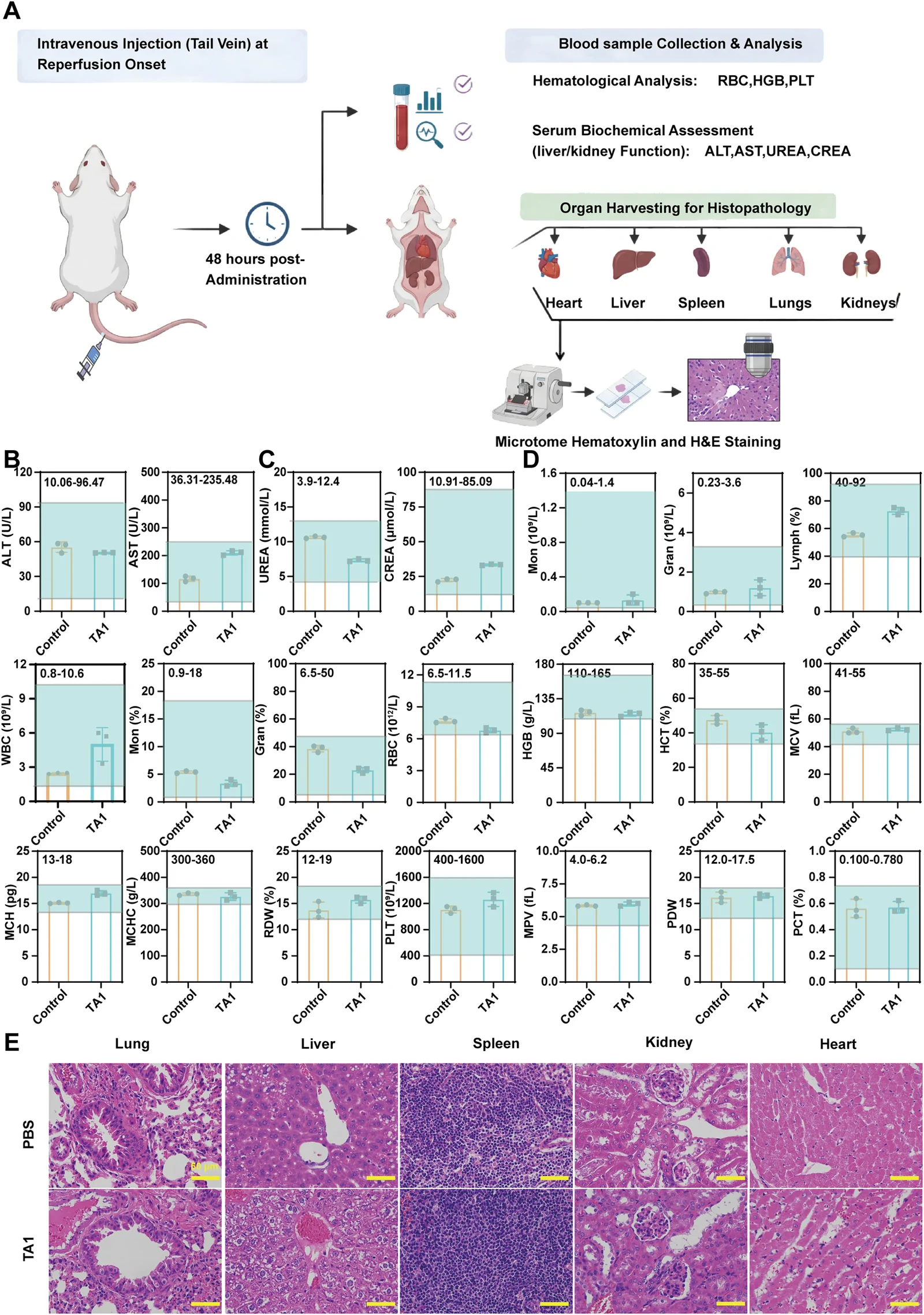
Assessment of hematology, liver and kidney function, and histopathology for **TA1**. **(A)** Biosafety evaluation was measured in the blood and major organs of mice 48 h after being intravenously injected with a single dose of **TA1** (136 μmol/kg). **(B,C)** Serum biochemical indexes for liver and kidney function, specifically ALT and AST **(B)**, and UREA and CREA **(C)**. **(D)** Comprehensive blood routine indexes, including RBC, HGB, PLT, and other related hematological parameters. **(E)** H&E staining of main organs (lung, liver, spleen, kidney, and heart) of mice after **TA1** (136 μmol/kg) administration. Scale bar = 50 μm. Data are expressed as mean ± SD (n = 3).

## 4 Discussion

Ischemic stroke represents a formidable clinical challenge characterized by a highly complex pathological network, necessitating therapeutic interventions capable of acting upon multiple molecular targets simultaneously (Cavalli et al., 2008; GBD, 2019 Stroke Collaborators, 2021; Tsao et al., 2023; Xue et al., 2025). In this study, we addressed the interdependent pathological loops of excitotoxicity and oxidative stress by rationally designing and synthesizing novel adamantane-nitrone hybrids (Viegas-Junior et al., 2007). Among them, compound **TA1** exhibited superior biological profiles compared to its analogs and the parent compound TBN. The findings comprehensively demonstrate that **TA1** ameliorates ischemic injury through robust *in vitro* cellular protection, *in vivo* reduction of cerebral infarct volume, and modulation of key signaling pathways governing oxidative stress, apoptosis, and neuroinflammation.

The molecular design strategy of **TA1** focused on hybridizing the nitrone-pyrazine pharmacophore of TBN—a well-documented free radical scavenger—with an adamantane scaffold (Sun et al., 2012b). Adamantane derivatives, such as memantine, are known to block excessive extracellular Ca^2+^ influx and mitigate excitotoxicity (Lipton, 2006). Our results validated this design rationale. In the t-BHP-induced oxidative damage model, **TA1** not only efficiently scavenged intracellular ROS and ONOO^−^ but also exhibited a more exceptional capacity to inhibit calcium overload than TBN at equivalent doses. Calcium overload is a critical upstream trigger that induces mitochondrial dysfunction and exacerbates oxidative stress. By effectively blunting this pathological calcium influx, **TA1** preserved mitochondrial structural and functional integrity, thereby interrupting the vicious cycle between excitotoxicity and oxidative stress more potently than the nitrone moiety alone.

Beyond maintaining cellular homeostasis, we explored the downstream molecular mechanisms underlying **TA1**’s neuroprotective effects. The Nrf2/HO-1 axis acts as a primary endogenous antioxidant defense system (Zhou et al., 2021; Qin et al., 2025). **TA1** treatment significantly upregulated Nrf2 and HO-1 expression in the ischemic penumbra, indicating a robust activation of antioxidant defenses that outperformed TBN. Furthermore, oxidative stress is inextricably linked to apoptosis and neuroinflammation (Manzanero et al., 2012; Liu et al., 2026). Ischemic injury fundamentally tilts the cellular balance toward programmed cell death (Yan et al., 2024) and activates NF-κB-mediated inflammatory cascades (Lo et al., 2005; Doyle et al., 2008). Our *in vivo* data clearly showed that **TA1** successfully restored the Bax/Bcl-2 balance, potently inhibited the executioner protein caspase-3, and suppressed the phosphorylation and nuclear translocation of p65 in the NF-κB pathway. These multidimensional regulatory effects—anti-apoptosis, enhanced antioxidant capacity, and anti-inflammation—provide a synergistic molecular mechanism that explains the substantial reduction in infarct size and the marked recovery of neurobehavioral functions observed in the MCAO mouse model.

A critical hurdle in developing neuroprotective agents for ischemic stroke is achieving sufficient penetration across BBB. The adamantane skeleton, characterized by its highly lipophilic cage structure, is frequently employed to improve the BBB permeability of centrally acting drugs (Wanka et al., 2013). Consistent with our hypothesis, the *in vitro* permeability assays demonstrated that **TA1** possessed superior transmembrane permeability (P_app_ = 6.73 × 10^−6^ cm/s) compared to TBN. Crucially, ischemic stroke frequently leads to the disruption of BBB integrity (Li et al., 2023), primarily driven by endothelial cell death. **TA1** mitigated OGD/R-induced damage in bEnd.3 cells and effectively repaired ischemia-induced BBB hyperpermeability. These findings provide preliminary *in vitro* evidence that **TA1** can cross an endothelial monolayer and preserve barrier integrity under OGD/R conditions, which may partly contribute to its observed *in vivo* efficacy. In addition, no overt abnormalities were detected in the selected hematological, serum biochemical, or histopathological indices at 48 h after a single intravenous dose of **TA1** (136 μmol/kg). Nevertheless, these findings should be interpreted as an initial assessment of short-term tolerability rather than a comprehensive evaluation of the pharmacokinetic or long-term safety profile of **TA1**.

Despite these promising findings, several limitations of the present study should be acknowledged. First, BBB permeability was evaluated using an *in vitro* bEnd.3 monolayer model. Although the P_app_ results provide preliminary evidence of endothelial permeability, they do not substitute for *in vivo* pharmacokinetic measurements or quantitative assessment of brain exposure. The plasma pharmacokinetics, time-dependent brain distribution, and exposure-response relationship of **TA1** therefore remain to be established. Second, the present study focused on acute efficacy and short-term tolerability following a single administration, with pathological, behavioral, and safety outcomes assessed within 24–48 h. Consequently, the durability of the functional benefits, repeated-dose tolerability, and potential long-term toxicity of **TA1** remain unknown. Dedicated longitudinal studies incorporating serial plasma and brain sampling, pharmacokinetic/pharmacodynamic analyses, extended behavioral follow-up, and repeated-dose safety assessments will be required to address these questions and more fully evaluate the translational potential of **TA1**.

In conclusion, by structurally fusing an adamantane moiety with a nitrone-pyrazine scaffold, this study identified **TA1** as a highly effective multi-target neuroprotective agent. By acting simultaneously to block calcium overload, scavenge reactive free radicals, protect mitochondrial function, and synergistically modulate the Nrf2/HO-1, Bax/Bcl-2, and NF-κB pathways, **TA1** successfully disrupts the ischemic cascade. With superior efficacy and BBB permeability compared to the lead compound TBN, **TA1** emerges as an innovative drug candidate with significant potential for the treatment of ischemic stroke.

## Data Availability

The original contributions presented in the study are included in the article/Supplementary Material, further inquiries can be directed to the corresponding authors.
