# Supplementary material for: Novel adamantane-nitrone hybrids: design, synthesis, and their multi-target neuroprotective effects against ischemic stroke
Source: Front Pharmacol. 2026 Oct 19;17:1974944. doi: 10.3389/fphar.2026.1974944 (PMC13724497; doi:10.3389/fphar.2026.1974944)
Supplement: Supplementary file 1 [file Supplementaryfile1.docx]

# Supporting Information

# 1. Supplementary figures

**Figure S1.**1H-NMR spectrum of TA1 in CDCl3

**Figure S2.**1H-NMR spectrum of TA2 in CDCl3

**Figure S3.**1H-NMR spectrum of TA3 in DMSO-d6

**Figure S4.**1H-NMR spectrum of TA4 in CDCl3

**Figure S5.**13C-NMR spectrum of TA1 in DMSO-d6.

**Figure S6.**13C-NMR spectrum of TA2 in DMSO-d6.

**Figure S7.**13C-NMR spectrum of TA3 in CDCl3.

**Figure S8.**13C-NMR spectrum of TA4 in DMSO-d6.

 **Figure S9.**HRMS spectrum of TA1.

**Figure S10.**HRMS spectrum of TA2.

**Figure S11.**HRMS spectrum of TA3.

**Figure S12.**HRMS spectrum of TA4.

**Figure S13.** HPLC chromatogram of TA1 (purity: 99.8%). Column: Agilent ZORBAX SB Aq-C18 (250 mm×4.6 mm×5 μm), Rate 1.0 mL/min; UV detection: 300 nm; Temperature: 30 ℃. The mobile phase consists of methanol and water containing 0.1% trifluoroacetic acid. The gradient program is as follows: 0-3 min, 20% methanol; 3-8 min, increased to 90%; 8-16 min, maintained at 90%; 16-20 min, decreased to 20%.

**Figure S14.** HPLC chromatogram of TA2 (purity: 98.5%). Chromatographic conditions are the same as described in Figure S13.

**Figure S15.** HPLC chromatogram of TA3 (purity: 99.6%). Chromatographic conditions are the same as described in Figure S13.

**Figure S16.** HPLC chromatogram of TA4 (purity: 99.3%). Chromatographic conditions are the same as described in Figure S13.
